# Open Distal Humerus Fracture With Brachial Artery Injury in Adults: A Case Report

**DOI:** 10.1155/cro/3366106

**Published:** 2025-12-07

**Authors:** Mohamad Omar Honeine, Youssef Jamaleddine, Elio Daccache, Chahine Assi, Fadi Hayek, Kaissar Yammine

**Affiliations:** ^1^ Department of Orthopedics and Traumatology, Lebanese American University Center-Rizk Hospital, Beirut, Lebanon; ^2^ Department of Vascular Surgery, Lebanese American University Center-Rizk Hospital, Beirut, Lebanon

**Keywords:** adult, brachial artery injury, distal humerus fracture, vascular repair

## Abstract

Penetrating trauma stands out as the predominant cause of vascular injury in the upper extremity, with the brachial artery being the vessel most frequently affected. While in the pediatric population, a traumatic injury to the brachial artery usually results from a distal humerus fracture; such scenarios are exceptionally reported in the adult population. We report the case of a 21‐year‐old woman who sustained a comminuted displaced distal humerus fracture associated with a weak radial pulse with no other ischemia signs. Only following open reduction and internal fixation did she present all signs of an acute ischemic limb. An emergency surgery showed a complex brachial artery injury over 3 cm of length. The vascular repair using a venous graft yielded a complete recovery of the patient. While brachial artery injury may be associated with distal humerus fractures in the pediatric population, this complication is exceedingly rare in adults. Our surgical case report serves as a call for clinicians to maintain a heightened awareness and suspicion for brachial artery injury, particularly in cases of severe elbow fractures. The absence of full signs of limb ischemia, owing to a rich collateral blood supply, underscores the importance of careful consideration and proactive management to address this serious complication.

## 1. Introduction

The upper extremity is most frequently subject to vascular injuries through penetrating trauma, with the brachial artery being the most commonly affected vessel [1]. Beyond the conventional causes, such as blunt and penetrating injuries, supracondylar fractures or dislocations of the humerus also pose a risk of brachial artery injury [2]. Even in cases where signs of limb ischemia are absent and patient findings are subtle, maintaining a heightened awareness for brachial artery injury is imperative, especially in instances of blunt trauma resulting from a fall onto the elbow and when signs indicating fracture or dislocation are present [3]. Approximately 12% of children experiencing a completely displaced supracondylar fracture are at risk of sustaining a blood vessel injury, with the brachial artery being the most commonly affected [4]. In the medical literature, scant articles have addressed the correlation between a distal humerus fracture and brachial artery injury in adults. One paper documented a simultaneous occurrence of arterial thrombosis [3], while another article detailed a pseudoaneurysm of the brachial artery following a proximal humerus fracture [5]. However, to our knowledge, there is no existing study that reports the association of a brachial artery tear with a distal humerus fracture in adults.

## 2. Case Presentation

**Figure 1:**
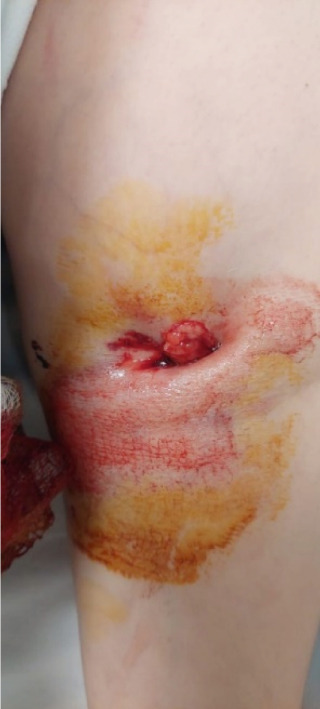
Open distal humerus fracture showing anterior wound in communication with the fracture site upon arrival.

**Figure 2:**
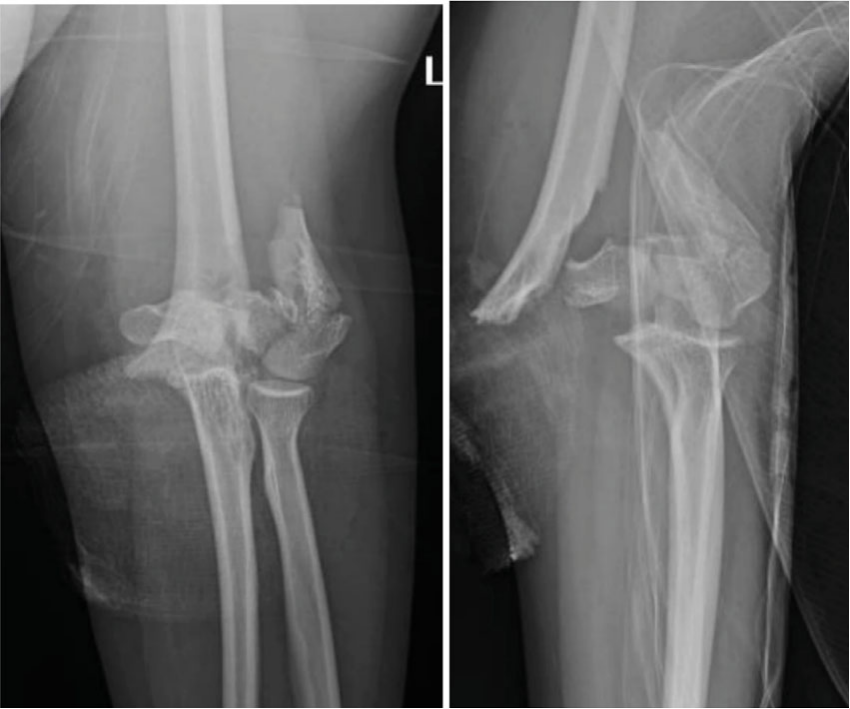
X‐rays of the patient showing a comminuted left distal humerus fracture, with joint line comminution, OTA classification Type C3.

We present the case of a 21‐year‐old female, previously in good health, who was brought to the emergency room following a motor vehicle accident, presenting with an open left distal humerus fracture. During the initial assessment, the patient exhibited a normal neurovascular exam in the left upper extremity, with palpable pulses, including the radial pulse, intact sensation, and distal motor function. Immediate administration of a tetanus shot and intravenous antibiotics was carried out, followed by immobilization of the left upper extremity after wound lavage and closure (Figure 1). X‐rays revealed a severe comminuted distal humeral fracture, classified as Type C3 according to the OTA classification, prompting an urgent decision for open reduction and internal fixation (Figure 2).

The patient underwent admission to the operating room, where prior to regional anesthesia, a reassessment of the neurovascular exam revealed the absence of a manually detected radial pulse, with only a faint pulse discernible through ultrasound. Subsequently, a vascular surgeon was consulted and recommended proceeding with open reduction and internal fixation, with reassessment planned after achieving reduction.

The patient was positioned in lateral decubitus with the left arm supported. A midline posterior incision allowed for soft tissue dissection, with the ulnar nerve identified, released, and safeguarded throughout the procedure. Employing a posterior approach, an olecranon osteotomy was performed to gain proper access to the fracture site.

**Figure 3:**
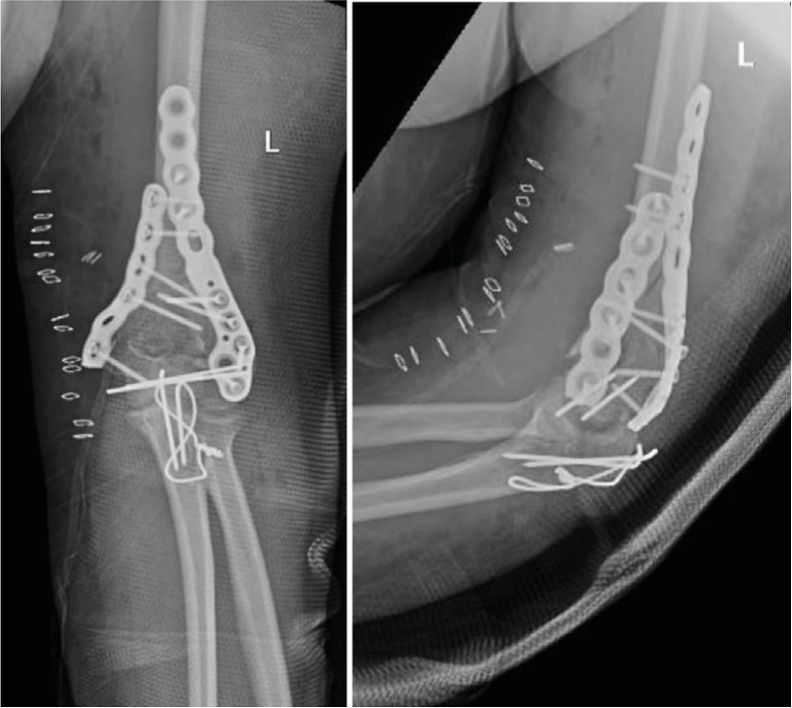
Postoperative x‐rays of the left elbow showing adequate reduction and fixation with two supracondylar humerus anatomic plates and fixation of the olecranon osteotomy.

Due to the significant comminution, fracture reduction posed considerable challenges. Initial internal fixation of the epiphysis was executed using k‐wires, followed by securing the reconstructed epiphysis to the diaphysis with two plates, placed both medially and laterally (Figure 3). Closure was performed with the use of a sump drain, and a posterior splint was applied.

**Figure 4:**
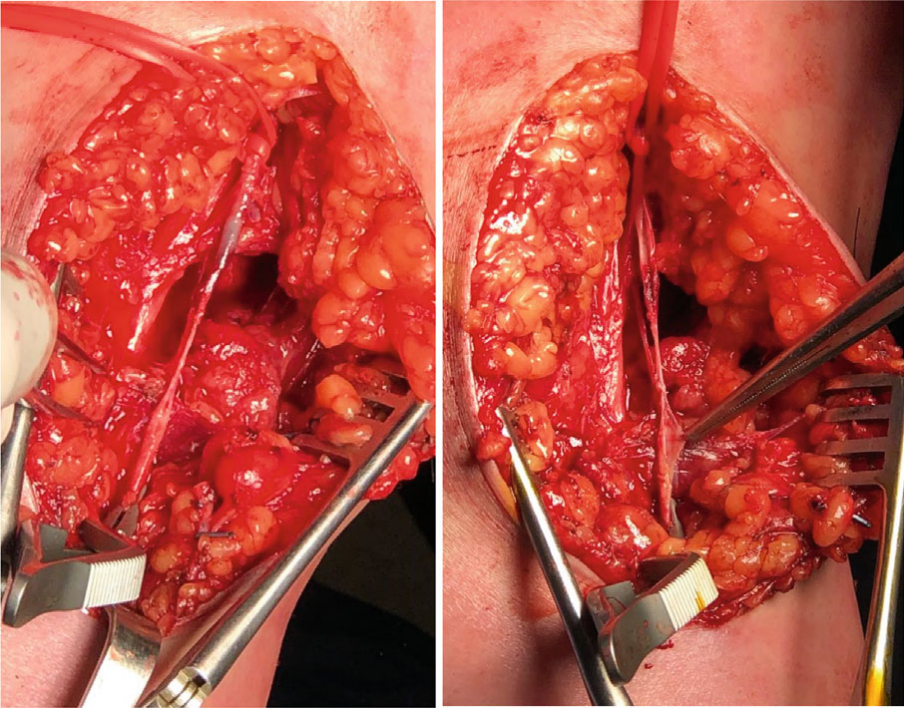
Intraoperative photo of the complex tear of the left brachial artery.

**Figure 5:**
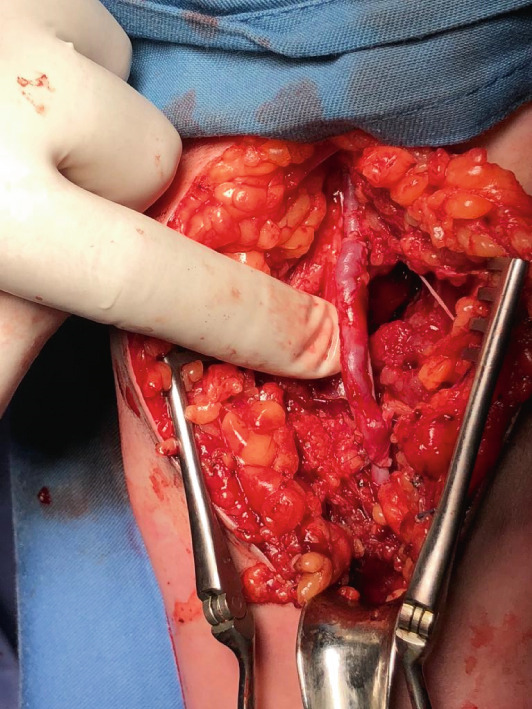
Arterial repair with a 5‐cm venous graft.

In the immediate postoperative period, a neurovascular assessment in the recovery room revealed a detectable radial pulse and a warm extremity, and subsequently, the patient was transferred to the surgical unit. On the following day, the patient reported severe pain in the left upper extremity that persisted despite opioid administration. Upon reexamination by the vascular team, an undetectable radial pulse was recorded both clinically and by US Doppler. Additionally, a prolonged capillary refill time of 7 s was noted, along with a cold, pulseless extremity. Consequently, the patient was promptly taken to the operating room. During exploration, a complex tear of 3 cm in the distal brachial artery was observed, presenting no possibility for direct repair. Subsequently, a vascular bypass was performed using a 5 cm graft harvested from the left greater saphenous vein (Figure 4). Postoperatively, the restoration of distal blood flow was confirmed, and the vascular assessment revealed a prominent radial pulse in the left upper extremity (Figure 5).

In the immediate second postoperative period, the patient reported a significant reduction in pain, with a visual analog scale (VAS) score of 5 compared to the preoperative score of 10. The neurological exam of the left upper limb demonstrated no sensation or motor deficit. The patient was discharged on Day 4 with a prescription for antibiotics for 10 days and a lifelong daily dose of aspirin 81 mg.

Weekly follow‐ups were conducted, and the splint was removed at 3 weeks, initiating elbow rehabilitation sessions. Over the course of 1 year post the initial accident, the patient maintained a normal neurovascular exam. However, at the last follow‐up, the patient exhibited a moderate limitation in the range of motion of her left elbow, with a flexion deficit of 20° and an extension deficit of 10°.

## 3. Discussion

This case report highlights an uncommon scenario where a limb‐threatening brachial artery injury was due to a distal humerus fracture in an adult individual. It has been documented that 5% of all open fractures are associated with vascular injuries necessitating repair, of which 16% result in amputation [6]. On the other hand, gunshot fractures in the distal humerus exhibit a notable occurrence of concurrent brachial artery injuries, approaching an incidence of 20% [7].

At its distal end, the brachial artery faces increased vulnerability, particularly when trapped between the bicipital aponeurosis and dislocated bony structures [3]. The likelihood of vascular compromise is associated with the force of energy transmitted to the tissues during the injury [8]. For instance, a higher frequency of brachial artery transections was documented in cases of open elbow dislocations and penetrating trauma compared to closed elbow dislocations and blunt trauma, owing to the involvement of high‐energy mechanisms [9]. Our case adds to the evidence with respect to these types of fractures; the anterior open wound would reflect the high‐energy trauma where the proximal fragment displaced anteriorly and ruptured the brachial artery before opening the skin.

Very few cases reported an injury of the brachial artery following arm and elbow trauma in adults. Fleischer et al. reported a brachial artery rupture associated with a compound elbow dislocation [10]. A late arterial complication has been reported following a supracondylar fracture in a 16‐year‐old person; an aneurysm of the brachial artery was diagnosed 10 years after the fracture with ulnar nerve entrapment as the clinical presentation [11].

The rich collateral blood supply may mask the signs of limb ischemia. Patients might present with subtle indicators, including palpable but diminished pulses, prolonged capillary refill time, and lower pulse oximetry readings [3]. Moreover, it is noteworthy that approximately 10% of confirmed arterial injuries may lack detectable changes in distal pulses or any evidence of ischemia [12]. In our case, we believe that the faint presence of the radial pulse before fracture fixation could be due to the anastomoses between the collaterals of the recurrent radial and ulnar arteries and the brachial artery. Furthermore, the vascular status needs to be checked repeatedly; our case showed that close monitoring of the radial pulse helped in keeping a high suspicion of arterial injury in the clinical agenda. The reappearance of a normal radial pulse following ORIF should not eliminate the diagnosis of a brachial artery injury. Our case demonstrated that a high degree of awareness should be given when recording an initial weak radial pulse and that the normalization of this clinical sign should not be relied on. This underscores the importance of comprehensive clinical evaluation, considering both overt and subtle manifestations, to accurately diagnose and address potential brachial artery injuries in such cases.

While there is no unanimous agreement on the optimal timing for repairing a vascular injury in relation to fracture management, the prevailing recommendation is to prioritize vascular repair beforehand to avert prolonged tissue ischemia [2]. Unstable fractures in the upper extremities pose a higher risk of blood vessel damage compared to those in the lower extremities, primarily due to the reduced surrounding muscle mass in the upper extremities [4]. Consequently, as observed in our case, our standard practice involves the routine fixation of the fracture before addressing vessel repair. This approach is aligned with the overarching goal of minimizing the risk of prolonged tissue ischemia and optimizing outcomes in such cases. The use of arteriography in patients with brachial artery injury was not standardized [13], and in particular in cases involving penetrating trauma [14]. However, its use has been steadily reported in the last decade, mainly the CT angiography [15], for the evaluation of upper limb vascular injuries as the gold standard investigation. The main indications for arteriography/CT angiography are diminished distal pulses and neurologic or muscular dysfunction while simultaneously appraising collateralization [16]. On the other hand, duplex ultrasonography emerges as a viable alternative, boasting a remarkable 99% sensitivity and 98% accuracy [15].

Various surgical techniques exist for repairing brachial artery injuries, encompassing lateral repair, end‐to‐end anastomosis, and interposition grafting, typically employing a saphenous vein graft [17]. The preference lies with end‐to‐end anastomosis, provided there is no tension or damage to major collateral vessels. In cases where this is not feasible, saphenous vein interposition grafting becomes the next best option [18], and this approach was adopted in our case.

This case report underscores the uncommon event of a brachial artery injury associated with a distal humerus fracture in adults. The complexities faced in managing such cases emphasize the importance for clinicians to meticulously assess the vascular status and intervene appropriately to ensure optimal patient outcomes.

## 4. Conclusion

This case report underscores the possibility of the association of a major artery injury and an open fracture of the distal humerus among the adult population. Identifying such cases can be challenging due to the extensive collateral blood supply. Notably, fractures in the distal humerus can manifest with damage to the distal portion of the brachial artery. We assert that this case report provides valuable insights for clinicians, emphasizing the importance of maintaining a heightened awareness when confronted with such situations, particularly when subtle indications of vascular injury are present.

## Consent

Consent was obtained by the patient to use his deidentified patient data in this case report for research purposes, including details about the course of his care, imaging, and laboratory results.

## Conflicts of Interest

The authors declare no conflicts of interest.

## Funding

No funding was received for this manuscript.

## Data Availability

The authors confirm that the data supporting the findings of this study are available within the article.
